# Coefficient of Variation in Metastatic Lymph Nodes Determined by ^18^F-FDG PET/CT in Patients with Advanced NSCLC: Combination with Coefficient of Variation in Primary Tumors

**DOI:** 10.3390/cancers16020279

**Published:** 2024-01-09

**Authors:** Sara Pellegrino, Rosa Fonti, Carlo Vallone, Rocco Morra, Elide Matano, Sabino De Placido, Silvana Del Vecchio

**Affiliations:** 1 Department of Advanced Biomedical Sciences, University of Naples “Federico II”, 80131 Naples, Italy; sara.pellegrino@unina.it (S.P.); rosa.fonti@unina.it (R.F.); carlovallone15@gmail.com (C.V.); 2 Department of Clinical Medicine and Surgery, University of Naples “Federico II”, 80131 Naples, Italy; rocco.mor4@gmail.com (R.M.); ematano@unina.it (E.M.); deplacid@unina.it (S.D.P.)

**Keywords:** non-small cell lung cancer, ^18^F-FDG PET/CT, coefficient of variation, heterogeneity of glycolytic phenotype, prognosis

## Abstract

**Simple Summary:**

Lung cancer is the leading cause of cancer-related death worldwide. The ^18^F-FDG PET/CT scan is used daily for the diagnosis and staging of lung cancer. The heterogeneity of ^18^F-FDG uptake inside tumor lesions may have prognostic implications for lung cancer patients. Therefore, we tested whether an index of heterogeneity such as the coefficient of variation (CoV), determined in both metastatic lymph nodes and primary tumors, can predict overall survival of lung cancer patients. We found that a combination of CoV of targeted lymph nodes with CoV of primary tumors in each patient provides a more accurate prognostic stratification of lung cancer allowing risk-adapted therapy in individual patients.

**Abstract:**

Purpose The aim of the present study was to test whether the coefficient of variation (CoV) of ^18^F-FDG PET/CT images of metastatic lymph nodes and primary tumors may predict clinical outcome in patients with advanced non-small cell lung cancer (NSCLC). Materials and Methods Fifty-eight NSCLC patients who had undergone ^18^F-FDG PET/CT at diagnosis were evaluated. SUVmax, SUVmean, CoV, MTV and TLG were determined in targeted lymph nodes and corresponding primary tumors along with Total MTV (MTV_TOT_) and Whole-Body TLG (TLG_WB_) of all malignant lesions. Univariate analysis was performed using Cox proportional hazards regression whereas the Kaplan–Meier method and log-rank tests were used for survival analysis. Results Fifty-eight metastatic lymph nodes were analyzed and average values of SUVmax, SUVmean, CoV, MTV and TLG were 11.89 ± 8.54, 4.85 ± 1.90, 0.37 ± 0.16, 46.16 ± 99.59 mL and 256.84 ± 548.27 g, respectively, whereas in primary tumors they were 11.92 ± 6.21, 5.47 ± 2.34, 0.36 ± 0.14, 48.03 ± 64.45 mL and 285.21 ± 397.95 g, respectively. At univariate analysis, overall survival (OS) was predicted by SUVmax (*p* = 0.0363), SUVmean (*p* = 0.0200) and CoV (*p* = 0.0139) of targeted lymph nodes as well as by CoV of primary tumors (*p* = 0.0173), MTV_TOT_ (*p* = 0.0007), TLG_WB_ (*p* = 0.0129) and stage (*p* = 0.0122). Using Kaplan–Meier analysis, OS was significantly better in patients with CoV of targeted lymph nodes ≤ 0.29 than those with CoV > 0.29 (*p* = 0.0147), meanwhile patients with CoV of primary tumors > 0.38 had a better prognosis compared to those with CoV ≤ 0.38 (*p* = 0.0137). Finally, we combined the CoV values of targeted lymph nodes and primary tumors in all possible arrangements and a statistically significant difference was found among the four survival curves (*p* = 0.0133). In particular, patients with CoV of targeted lymph nodes ≤ 0.29 and CoV of primary tumors > 0.38 had the best prognosis. Conclusions The CoV of targeted lymph nodes combined with the CoV of primary tumors can predict prognosis of NSCLC patients.

## 1. Introduction

Lung cancer is one of the most frequently diagnosed cancers and the leading cause of cancer-related deaths worldwide [1]. Non-small cell lung cancer (NSCLC) accounts for about 80% of all lung malignancies and includes several histological subtypes such as adenocarcinoma, squamous cell and large cell carcinoma [2]. Despite the significant improvement in our understanding of disease biology and molecular pathways leading to refinement of therapeutic strategies, the prognosis of lung cancer patients with advanced disease still remains poor. In fact, after an initial response to therapeutic regimens, tumors become resistant to treatment and this will invariably cause disease progression and death [3]. The occurrence of resistance is usually ascribed to the expansion of tumor cell subclones with different molecular and genetic profiles, resulting in tumor heterogeneity [4].

Several genetic, epigenetic and non-genetic mechanisms can cause heterogeneity in both primary lung tumors and metastatic lesions [5,6,7]. For instance, metastatic lesions at different body sites can derive from distinct cellular populations inside the primary tumor, resulting in intermetastatic heterogeneity [8]. Overcoming tumor heterogeneity is one of the main challenges in the development of personalized treatment strategies in NSCLC patients.

Recently, tumor heterogeneity of diagnostic images has been evaluated by texture analysis of computed tomography (CT), magnetic resonance (MR) and positron emission tomography/CT (PET/CT) images. By measuring the spatial distribution and variations of the signal on a voxel-by-voxel basis [9,10], texture analysis allows to obtain subvisual quantitative information [11,12] that can have predictive and prognostic significance.

By analyzing ^18^F-Fluorodeoxyglucose (^18^F-FDG) PET/CT images obtained in NSCLC patients, previous studies have shown that tumor heterogeneity of ^18^F-FDG uptake is correlated to intratumoral histopathologic heterogeneity in these patients [13,14]. Moreover, recent studies have demonstrated that texture analysis of ^18^F-FDG PET/CT images, by reflecting tumor metabolic heterogeneity, is associated with clinical outcomes in NSCLC patients [15,16,17].

The PET-based coefficient of variation (CoV) is a first-order texture feature indicating the heterogeneity of the glycolytic phenotype within the metabolic tumor volume and it is determined from standard deviation (SD) divided by SUVmean. The advantage of using this parameter as an index of heterogeneity is that its extraction from images is easy and does not require sophisticated software. In a previous study, Pahk et al. [18] determined CoV values in ^18^F-FDG PET/CT images of primary lung tumors and correlated them to the presence of lymph node metastases in patients with clinically suspected N2. In particular, CoV values of primary tumors were significantly higher in patients with pathologically confirmed positive mediastinal lymph nodes as compared to the group of patients with negative mediastinal lymph nodes. We have previously studied patients with non-small cell lung cancer who underwent ^18^F-FDG PET/CT scan before any therapy and found that patients with CoV values of primary tumors lower than the threshold had significantly poorer overall survival (OS) as compared to those with CoV values higher than the cut-off [19]. The aim of the present study was to test whether CoV values determined in ^18^F-FDG positive lymph nodes can improve the prediction of clinical outcome of NSCLC patients.

## 2. Materials and Methods

### 2.1. Patients 

^18^F-FDG PET/CT studies were performed in 58 patients (39 men, 19 women; mean age 64 ± 11 years; range 38–83 years) enrolled with the following inclusion criteria: histologically proven non-small cell lung cancer; stage III and IV disease; whole-body ^18^F-FDG PET/CT scan performed at our Institution before any therapy; simultaneous presence of primary tumor and metastatic lymph node suitable for segmentation (SUVmax > 2.5) in each patient. The exclusion criteria were: prior lung or chest malignancy; prior chemotherapy or chest radiotherapy; no pathological diagnosis on primary lung lesion; unsuitable simultaneous segmentation of primary tumor and metastatic lymph node in the same patient; missing imaging data for analysis; missing clinical follow-up. 

All ^18^F-FDG PET/CT scans were performed before any therapy. The clinical characteristics, histology, stage and therapy after ^18^F-FDG PET/CT scan are summarized in Table 1. 

The institutional Ethics Committee approved this retrospective study (Protocol N. 352/18) and all patients signed an informed consent form. 

Based on their age, stage, tumor histology, molecular pathology, PD-L1 expression, performance status and comorbidities [20], 48 patients received chemotherapy after an ^18^F-FDG PET/CT scan, in association with radiotherapy in 2 patients and combined with immunotherapy in 15 patients 2. No specific cancer treatment was administered to the remaining 10 patients due to advanced age or severe comorbidities. 

The mean follow-up period was 12 months (range 1–43 months). Progression-free survival (PFS) included the interval between the date of the ^18^F-FDG PET/CT scan performed at diagnosis and the first observation of progressive disease, relapse or death. OS was determined from the date of the ^18^F-FDG PET/CT scan performed at diagnosis to the date of death.

### 2.2. ^18^F-FDG PET/CT Study and Image Analysis

The protocol of the ^18^F-FDG PET/CT scan has been reported in detail elsewhere [19]. Briefly, ^18^F-FDG (370 MBq) was i.v. injected into patients after 8 hours of fasting and the scan was acquired 60 minutes after tracer administration with an Ingenuity TF scanner (Philips Healthcare, Best, The Netherlands). Co-registered CT images were obtained using 120 kV, 80 mAs, 0.8 s rotation time, pitch of 1.5 whereas whole body PET scan was acquired in 3-dimensional mode using 3 min per bed position. After iterative reconstruction, transaxial, sagittal and coronal images were obtained and preliminarily examined using Ingenuity TF software (IntelliSpace Portal V5.0)

PET/CT images were then analyzed on a different workstation using the LIFEx program [21]. Focal ^18^F-FDG uptake in primary lesions and involved lymph nodes was identified and subjected to segmentation. This analysis was performed on the metastatic regional lymph node with the highest SUVmax value and on the corresponding primary tumor that was located in the left (38%) or right (62%) lung. A volume of interest (VOI) of each targeted lymph node and corresponding primary tumor was automatically delineated on PET images by setting an absolute threshold for SUV at 2.5 [22,23]. Co-registered CT images were used to assess the accuracy of lesion segmentation. Then, SUVmean, CoV, SUVmax, metabolic tumor volume (MTV) and total lesion glycolysis (TLG) were obtained for targeted lymph nodes and primary tumors. In particular, the CoV was determined as Standard Deviation (SD) divided by SUVmean. The size of voxels of PET images was 4 mm × 4 mm × 4 mm. MTV of each lesion was determined by grouping all spatially connected voxels with a SUV higher than the threshold of 2.5. TLG was determined by multiplying MTV of each lesion for the corresponding SUVmean. All these parameters derived from the involved lymph node with the highest SUVmax value in each patient and corresponding primary lesions were included in the survival analysis. 

The same segmentation procedure was then extended to all metabolically active lesions for determination of total metabolic tumor volume (MTV_TOT_) and whole-body total lesion glycolysis (TLG_WB_). These parameters were calculated by the sum of MTV or TLG values obtained from each primary tumor, all involved lymph nodes and distant metastatic lesions of each patient [24]. Coalescent lymph nodes were considered as a single lesion. Due to the high physiological FDG uptake in the cerebral cortex, brain metastases were excluded from the analysis.

### 2.3. Statistical Analysis

The software MedCalc for Windows, version 10.3.2.0 (MedCalc Software, Mariakerke, Belgium) was used for statistical analysis. The best discriminative values of imaging parameters between patients who had died and survivors, as well as between patients with and without progression disease, were determined by receiver-operating characteristic (ROC) curves. Clinical and imaging variables were subjected to univariate and multivariate analyses using Cox proportional hazards regression whereas the Kaplan–Meier method and log-rank tests were used for survival analysis. A statistically significant result was indicated by a *p* value < 0.05.

## 3. Results

**Figure 1:**
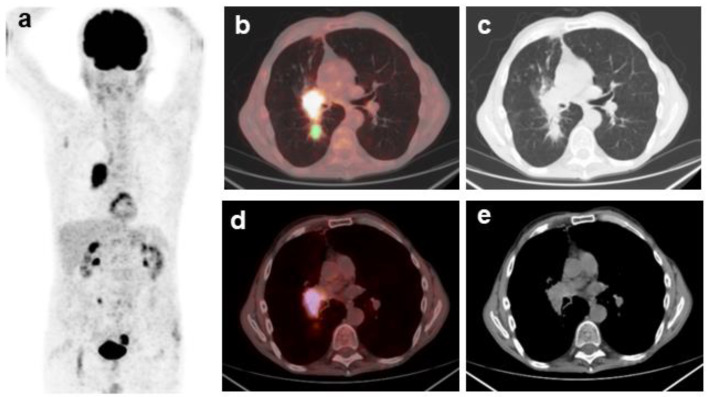
Representative images of ^18^F-FDG PET/CT scan in a patient with stage IIIA non-small cell lung cancer. (**a**). Maximal intensity projection PET image; (**b**). transaxial fusion image of co-registered PET and CT showing high FDG uptake in the primary lung tumor and hilar lymph node. A tridimensional region of interest was drawn around the primary lung tumor (green); (**c**). corresponding CT image of the thorax using window for lung parenchyma visualization; (**d**). transaxial fusion image of co-registered PET and CT showing the FDG avid metastatic hilar lymph node. A tridimensional region of interest was drawn around the targeted lymph node (pink); (**e**). corresponding CT image of the thorax using window for mediastinal visualization.

^18^F-FDG PET/CT scans were analyzed and imaging parameters were extracted from metastatic lymph nodes and primary tumors. Figure 1 shows representative images of the VOIs drawn around the targeted lymph node and primary tumor in a patient with stage IIIA NSCLC.

Mean values (±SD) of SUVmax, SUVmean and CoV derived from lymph nodes were 11.89 ± 8.54, 4.85 ± 1.90 and 0.37 ± 0.16 whereas the same parameters determined in primary tumors were 11.92 ± 6.21, 5.47 ± 2.34 and 0.36 ± 0.14 (Table 2). 

In addition, mean values of MTV and TLG for lymph nodes were 46.16 ± 99.59 mL and 256.84 ± 548.27 g and for primary tumors they were 48.03 ± 64.45 mL and 285.21 ± 397.95 g, respectively (Table 2). Then, the volumetric imaging parameters MTV_TOT_ and TLG_WB_, that reflected the whole-body tumor burden, were calculated by summing MTV and TLG values of all measurable lesions detected in each patient. In particular, a total of 364 lesions including 58 primary tumors, 174 lymph nodes and 132 distant metastases were analyzed to obtain the whole metabolic tumor burden of each patient. Mean MTV_TOT_ and TLG_WB_ values were 157.75 ± 166.71 mL and 887.49 ± 1038.44 g, respectively.

Patients’ survival was evaluated after a mean follow-up of 12 months. Among the 58 patients, 37 had progression and died, 10 had progression and survived while 11 patients had stable disease. The best discriminative values for SUVmax, SUVmean and CoV of targeted lymph nodes and primary tumors between patients who had died and survivors were determined by ROC curve analysis. Supplementary Materials Figure S1 shows ROC curves for CoV of metastatic lymph nodes and CoV of primary tumors. The thresholds for SUVmax, SUVmean and CoV of targeted lymph nodes were 7.3, 4.4 and 0.29, respectively, whereas for primary tumors, thresholds for the same parameters were 13, 4.7 and 0.38, respectively.

Age, gender, histology, stage, imaging parameters derived from targeted lymph nodes and primary tumors (SUVmax, SUVmean, CoV, MTV and TLG) and whole-body volumetric parameters (MTV_TOT_ and TLG_WB_) were included in univariate analysis and results are reported in Table 3. 

**Figure 2:**
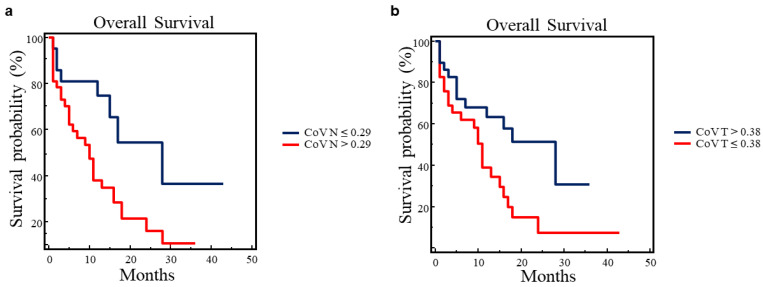
Overall survival analysis in 58 patients with advanced NSCLC. (**a**). OS was evaluated by Kaplan–Meier analysis and log-rank test using the cut-off value of CoV derived from targeted lymph nodes (CoV N = 0.29). A significantly prolonged OS was observed in patients having CoV N ≤ 0.29 (χ^2^ = 5.9570, *p* = 0.0147); (**b**). OS was evaluated by Kaplan–Meier analysis and log-rank test using the cut-off value of CoV derived from primary tumors (CoV T = 0.38). A significantly prolonged OS was observed in patients having CoV T > 0.38 (χ^2^ = 6.0795, *p* = 0.0137).

OS was predicted by SUVmax (*p* = 0.0363), SUVmean (*p* = 0.0200) and CoV (*p* = 0.0139) of targeted lymph nodes and, as previously reported, by CoV of primary tumors (*p* = 0.0173), MTV_TOT_ (*p* = 0.0007), TLG_WB_ (*p* = 0.0129) and stage (*p* = 0.0122). Kaplan–Meier analysis and long-rank testing performed using the threshold of SUVmax and SUVmean of targeted lymph nodes showed that patients with SUVmax ≤ 7.3 had significantly better OS as compared to those with SUVmax > 7.3 (χ^2^ = 4.3823, *p* = 0.0363), whereas patients with SUVmean ≤ 4.4 showed a better OS than patients having SUVmean > 4.4 (χ^2^ = 5.8865, *p* = 0.0153). Interestingly, the CoV of lymph nodes and primary tumors predicted OS in opposite directions (Figure 2).

In fact, Kaplan–Meier analysis and long-rank testing showed that patients with CoV of targeted lymph nodes ≤ 0.29 had significantly better OS as compared to patients having CoV > 0.29 (χ^2^ = 5.9570, *p* = 0.0147) (Figure 2a), whereas patients with CoV of primary tumors > 0.38 showed a prolonged OS compared to patients with CoV ≤ 0.38 (χ^2^ = 6.0795, *p* = 0.0137) (Figure 2b). 

All statistically significant variables were then tested in the multivariate analysis of OS and only CoV of primary tumors and MTV_TOT_ were retained in the model (χ^2^ = 16.6320, *p* = 0.0002). 

**Figure 3:**
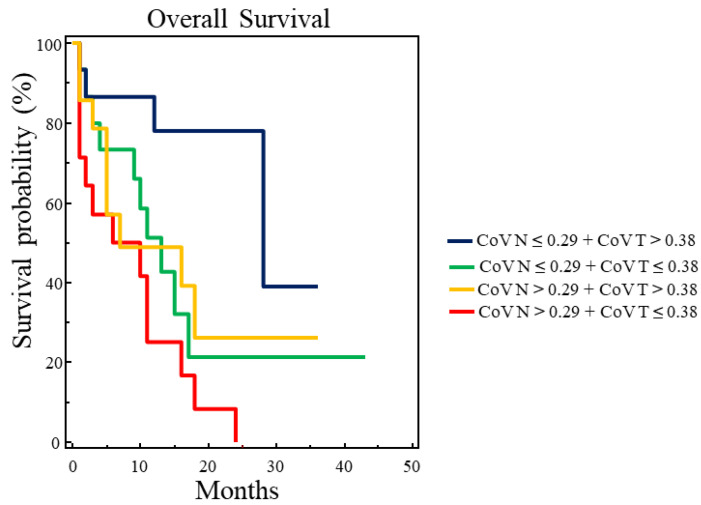
Overall survival analysis in 58 patients with advanced NSCLC using all possible combinations of CoV derived from targeted lymph nodes (CoV N) and CoV derived from primary tumors (CoV T). A statistically significant difference was found among the four survival curves (χ^2^ = 10.7245, *p* = 0.0133). In particular, patients with CoV N ≤ 0.29 and CoV T > 0.38 showed the best OS with a median survival time of 28 months.

Although CoV of targeted lymph nodes was not an independent prognostic factor for OS, we combined it with the CoV value determined on the corresponding primary tumor and tested whether the four possible combinations could better stratify our population. A statistically significant difference among the four survival curves (χ^2^ = 10.7245, *p* = 0.0133) was found via Kaplan–Meier analysis using the combined thresholds of CoV. In particular, patients with CoV of targeted lymph nodes ≤ 0.29 and CoV of primary tumors > 0.38 had the best prognosis (median OS = 28 months) (Figure 3).

**Figure 4:**
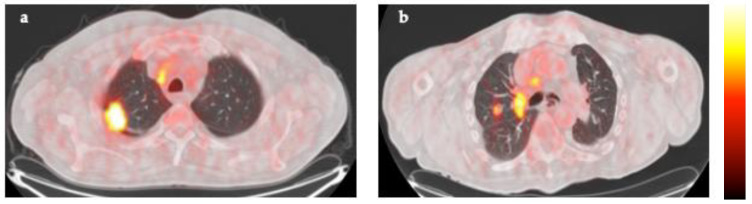
Representative ^18^F-FDG PET/CT fusion images of a patient of the best prognosis group (**a**), based on the combination of CoV N ≤ 0.29 with CoV T > 0.38, and a patient of the worst prognosis group (**b**), based on the combination of CoV N > 0.29 with CoV T ≤ 0.38. The color bar indicates ^18^F-FDG uptake.

Figure 4 shows representative ^18^F-FDG PET/CT images of patients with the best prognosis, having CoV of targeted lymph nodes ≤ 0.29 and CoV of primary tumors > 0.38 (Figure 4a), and patients with the worst prognosis, having CoV of targeted lymph nodes > 0.29 and CoV of primary tumors ≤ 0.38 (Figure 4b).

When considering PFS, the best discriminative values between patients with and without progression were 7.3, 5 and 0.29 for SUVmax, SUVmean and CoV of targeted lymph nodes, respectively, whereas thresholds of the same parameters of primary tumors were 8.8, 4.7 and 0.35, respectively. At univariate analysis, PFS was significantly predicted by SUVmax (*p* = 0.0464) and SUVmean (*p* = 0.0223) of targeted lymph nodes, CoV of primary tumors (*p* = 0.0369) and MTV_TOT_ (*p* = 0.0089). These variables along with CoV of targeted lymph nodes, stage and TLG_WB_ were tested in multivariate analysis and CoV of primary tumors and MTV_TOT_ were retained in the model for the prediction of PFS (χ^2^ = 11.8320, *p* = 0.0027).

## 4. Discussion

The present study showed that the heterogeneity of glycolytic phenotype within both metastatic lymph nodes and primary lung tumors assessed by PET-based coefficient of variation can predict clinical outcome in NSCLC patients. In particular, OS was significantly worse in patients with a CoV of targeted lymph nodes higher than the cut-off value than those having a CoV lower than the threshold. Furthermore, in agreement with our previous findings [19], patients with a CoV of primary tumors lower than the cut-off value had significantly worse OS as compared to patients with a CoV higher than the threshold. Therefore, the CoV of targeted lymph nodes and CoV of primary tumors are able to predict OS in NSCLC patients in opposite directions. These findings may be explained by the fact that heterogeneity of tracer uptake in primary tumors derives mainly from the presence of tumor cells lacking the glycolytic phenotype dispersed in the population of ^18^F-FDG avid malignant cells, whereas in metastatic lymph nodes, heterogeneity is mainly due to the presence of tumor cells having a glycolytic phenotype in the normal lymph node structure.

A prospective study conducted by Carvalho et al. [25] evaluated the prognostic role of texture features of PET images derived from both primary tumors and lymph nodes in NSCLC patients in stages I-III. In agreement with our findings, they reported that combining radiomic features derived from involved lymph nodes with those obtained from primary tumors significantly improves the prognostic stratification of NSCLC patients. Furthermore, previous studies have evaluated whether the heterogeneity of ^18^F-FDG uptake can predict malignancy of suspected mediastinal lymph nodes in NSCLC patients. In particular, Hua et al. [26] reported that CoV was significantly higher in metastatic lymph nodes than in healthy lymph nodes. Moreover, Budiawan et al. [27] reported that ^18^F-FDG uptake is more heterogeneous in metastatic lymph nodes than in inflammatory lymph nodes as assessed by CoV in NSCLC patients. These findings are in agreement with our results indicating that higher CoV values of targeted lymph nodes by revealing the presence of malignant tumor cells predicted a worse OS in NSCLC patients. 

A potential limitation of our study could be the unbalanced distribution of histological subtypes in the study population with a prevalence of adenocarcinomas. In fact, a different composition of the study population may lead to different thresholds values for the CoV of targeted lymph nodes and CoV of primary tumors. However, the predictivity of the two parameters in the opposite direction and the advantage of their combination will be likely confirmed in a further study including a significant number of all histological subtypes. 

The identification of prognostic features by texture analysis is a very effective approach for the stratification of lung cancer patients [28,29,30], despite the fact that many of these features do not have a real biological meaning. By examining the prognostic role of CoV in involved lymph nodes and primary tumors, we realized that changing the lesion site may reverse the biological meaning of heterogeneity of a given image feature. In fact, we should consider that the heterogeneity evaluated on ^18^F-FDG PET images by texture analysis refers to the spatial variability of tracer uptake and hence to a variable combination of tumor, inflammatory, immune and normal cells showing different glucose consumption rate. Therefore, the first question that needs to be asked to understand results of texture analysis is whether the signal has a favorable or unfavorable prognostic significance and then whether the heterogeneity results from the addition of the signal (as for lymph nodes) or subtraction of signal (as in primary tumors). In agreement with these observations, high CoV values in metastatic lymph nodes indicate poor prognosis, whereas high CoV values in primary tumors correlate with good prognosis. Our study highlighted the different prognostic behavior of the same texture variable when extracted from primary tumors or from metastatic lymph nodes. This may significantly improve the interpretation of prognostic texture variables and their implementation in appropriate clinical contexts. Furthermore, our findings indicate that texture analysis should be performed separately on primary tumors, involved lymph nodes and metastatic lesions of cancer patients and then results may be eventually combined using appropriate models. 

## 5. Conclusions

The CoV of targeted lymph nodes and CoV of primary tumors are able to predict the clinical outcomes of NSCLC patients with advanced disease in opposite directions. This simple first-order parameter can be easily determined on PET images, thus providing information on the expression variability of glycolytic phenotype in both metastatic and primary lesions.

## Figures and Tables

**Table 1:** Clinical characteristics, histological subtypes, stage and treatment of 58 patients with advanced NSCLC.

Characteristic	N°
**Patients**	58
**Age**	
Mean ± SD	64 ± 11
Range	38–83
**Gender**	
Male/Female	39/19
**Histology**	
Adenocarcinoma	31
Squamous cell carcinoma	13
Large cell carcinoma	3
Not otherwise specified	11
**TNM stage**	
III (A/B/C)	16 (2/9/5)
IV (A/B)	42 (10/32)
**Treatment after ^18^F-FDG PET/CT**	
Chemotherapy	31
Chemoradiotherapy	2
Chemotherapy/Immunotherapy	15
No cancer therapy	10

SD, Standard Deviation.

**Table 2:** PET-based imaging parameters obtained by ^18^F-FDG PET/CT analysis of 58 targeted lymph nodes and 58 primary tumors.

Parameters	Mean ± SD	Range
**SUVmax**		
Lymph nodes	11.89 ± 8.54	3.53–46.82
Primary tumors	11.92 ± 6.21	3.05–38.51
**SUVmean**		
Lymph nodes	4.85 ± 1.90	2.94–12.32
Primary tumors	5.47 ± 2.34	2.71–16.37
**CoV**		
Lymph nodes	0.37 ± 0.16	0.10–0.86
Primary tumors	0.36 ± 0.14	0.07–0.66
**MTV (mL)**		
Lymph nodes	46.16 ± 99.59	0.45–514.45
Primary tumors	48.03 ± 64.45	0.26–321.22
**TLG (g)**		
Lymph nodes	256.84 ± 548.27	1.63–3221.44
Primary tumors	285.21 ± 397.95	0.69–2244.66

SD, standard deviation; CoV, coefficient of variation; MTV, metabolic tumor volume; TLG, total lesion glycolysis.

**Table 3:** Predictors of overall and progression-free survival by univariate analysis of clinical variables and imaging parameters of targeted lymph nodes (N) and primary tumors (T).

Variable	Overall Survival		Progression-Free Survival	
	χ^2^	*p*	χ^2^	*p*
Age	0.1450	0.7033	0.2390	0.6251
Gender	0.1650	0.6845	1.0990	0.2945
Histology	2.3070	0.1288	1.2440	0.2646
SUVmax N	4.3810	0.0363	3.9680	0.0464
SUVmean N	5.4100	0.0200	5.2210	0.0223
CoV N	6.0550	0.0139	3.3430	0.0675
SUVmax T	1.8130	0.1782	1.6430	0.1999
SUVmean T	2.0720	0.1500	3.0300	0.0818
CoV T	5.6640	0.0173	4.3560	0.0369
Lymph node MTV	0.8220	0.3645	1.6470	0.1993
Lymph node TLG	0.8510	0.3562	1.7760	0.1826
Primary tumor MTV	2.2380	0.1347	1.4700	0.2254
Primary tumor TLG	1.3280	0.2492	1.0260	0.3111
MTV_TOT_	11.5750	0.0007	6.8410	0.0089
TLG_WB_	6.1870	0.0129	3.5400	0.0599
Stage	6.2770	0.0122	2.9570	0.0855

CoV, coefficient of variation, MTV, metabolic tumor volume; TLG, total lesion glycolysis*;* MTV_TOT_, total metabolic tumor volume; TLG_WB_, whole-body total lesion glycolysis.

## Data Availability

The datasets used and/or analyzed during the current study are available from the corresponding author on reasonable request.

## Supplementary Materials

The following supporting information can be downloaded at: https://www.mdpi.com/article/10.3390/cancers16020279/s1, Figure S1: ROC curves for CoV of metastatic lymph nodes (CoV N) and CoV of primary tumors (CoV T).
