# Supplementary material for: Coefficient of Variation in Metastatic Lymph Nodes Determined by 18F-FDG PET/CT in Patients with Advanced NSCLC: Combination with Coefficient of Variation in Primary Tumors
Source: Cancers (Basel). 2024 Jan 9;16(2):279. doi: 10.3390/cancers16020279 (PMC10813913; doi:10.3390/cancers16020279)
Supplement: Supplementary file 1 [file cancers-16-00279-s001.zip › cancers-2784233-supplementary.pdf]

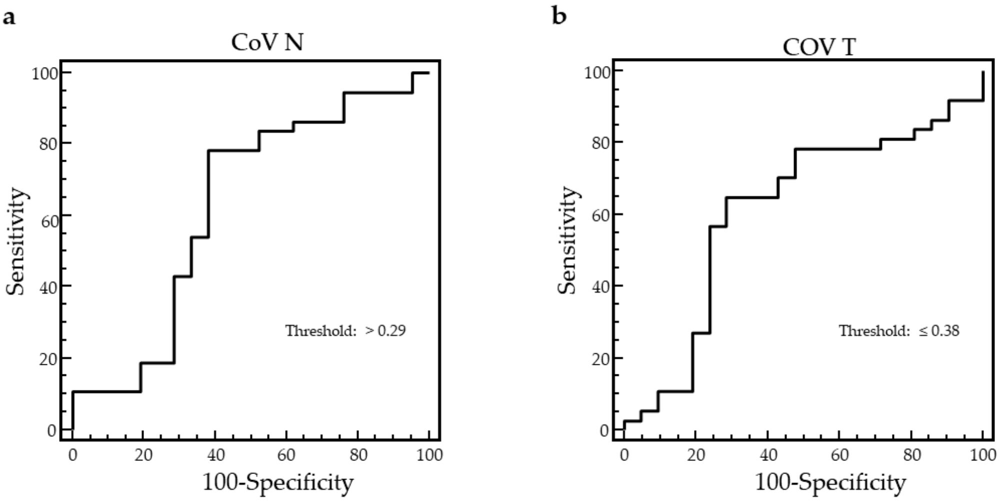

Supplementary Figure S1: ROC curves for CoV of metastatic lymph nodes (CoV N) and CoV of primary tumors (CoV T). ROC curve analysis showed that the best discriminative values for CoV N and CoV T between patients who had died and survivors were 0.29 (**a**) and 0.38 (**b**), respectively.
